# A 6 year Geohelminth infection profile of children at high altitude in Western Nepal

**DOI:** 10.1186/1471-2458-8-98

**Published:** 2008-03-27

**Authors:** Chiranjay Mukhopadhyay, Godwin Wilson, Kiran Chawla, Binu VS, PG Shivananda

**Affiliations:** 1 Department of Microbiology, Kasturba Medical College, Manipal University, Karnataka, India; 2 Laboratory medicine and pathology, Al-Khor Hospital, Hamad Medical Corporation, Doha, Qatar; 3 Department of Microbiology, Kasturba Medical College, Manipal University, Karnataka, India; 4 Department of Statistics, Manipal University, Karnataka, India; 5 Department of Microbiology, Sikkim Manipal Institute of Medical Sciences, Gangtok, Sikkim, India

## Abstract

**Background:**

Geohelminth infections are a major problem of children from the developing countries. Children with these infections suffer from developmental impairments and other serious illnesses. This study aimed to measure the prevalence of geohelminth infection, infection intensity as well as the change in the intensity in children from Western Nepal over years.

**Methods:**

This 6-year hospital based prospective study at the Manipal Teaching Hospital, Pokhara included children (< 15 years) visiting the hospital from Kaski and 7 surrounding districts. Samples were also collected from children in the community from different medical camps. Three stool samples from every child were processed using direct and concentration methods. The Kato-Katz technique was used for measuring the intensity of infection.

**Results:**

The overall prevalence in hospital - attending children was 9.2% with 7.6% in preschool (0 – 5 y) and 11.0% in school-age (6 – 15 y) children, and in community 17.7% with 14.8% in pre-school and 20.5% in school-age children. *Ascaris lumbricoides*, *Trichuris trichiura*, *Ancylostoma deodenale *and *Strongyloides stercoralis *were the common geohelminths with a gradual decrease in worm load over the years. School-age children were found to be significantly more prone to geohelminth infection as compared to preschool children, but no statistical difference was detected by gender, district as well as season.

**Conclusion:**

This heavy infection of geohelminths in children should be corrected by appropriate medication and maintaining strict personal hygiene. Health education, clean water, good sewage management and a congenial environment should be ensured to minimise infection.

## Background

Geohelminth infections are a major problem in developing countries of the South-east Asian region [1,2], yet there is little information for most of the countries that describes the intensity of the problem. There are relatively simple but effective control strategies for these widespread diseases which, unfortunately, are often overlooked. The public health burden of these helminth infections has been consistently underestimated, although school-age children are at highest risk and may suffer from nutritional deficits, cognitive impairments, serious illnesses, and in occasional cases, death [2-8]. The risk of the individuals suffering geohelminth infection-related morbidity appears to be a joint function of the number of species harboured and/or the infection intensity of any species [9,10].

Geohelminth infection prevalence rate of nearly 100% has been reported from local population in Nepal [11-13], whereas the annual prevalence rate among rural population during years 1985–92 was documented within the range of 18.0–36.6% with a marginal decrease in successive years [14]. The incidence, however, showed an increase after the year 1992. A baseline parasitological survey (supported by the World Food Programme) in 1996 from Parsa and Dailekh districts in Nepal among 711 school children (mean age 9.266), showed an overall prevalence of 90% with *Ascaris lumbricoides *21.9%, *Trichuris trichiura *19.2% and hookworm 64.7%. Moderate to heavy infection was seen in 20% of the children. Boys were more infected than girls [15].

The inhabitants of Kaski and the surrounding districts like Lamjung, Manang, Myagdi, Baglung, Parbat, Shangha and Tanahu use tap water as well as river/lake water for drinking and household purposes. Some houses are pucca (made of brick and cement) with attached bathroom. Health control measures like tap water supply, deworming programmes of school-age children, decontamination of well water sources have been started but have failed to protect against exposure to environmental health risks due to frequent supply of unsafe drinking water, inadequate sanitation and excreta disposal, poor drainage, inadequate solid waste removal, lack of animal control, and improper protection of catchment area which have all been identified as important risk factors in the transmission of geohelminth infection [16,17]. Some home-based water treatment systems with filtration, flocculation and safe sealed storage containers are used by the local people. Lack of hygienic behaviour by children and adults clearly increase the risk of geohelminth infection [18,19]. Home gardens are a source of income generation as well as a regular source of fruits and vegetables in this part, where night soil is used as manure and most of the people work bare-foot [20]. This makes them more susceptible to geohelminthiasis.

The aim of this study is to report the prevalence and intensity of geohelminths in children, and its association with seasonal variation in Western Nepal.

## Methods

Pokhara the capital city of Kaski district (one of the seventy-five districts of Nepal), Gandaki zone, is located in the western part of Nepal at an altitude of 2,713 feet/827 m above sea level. The annual rainfall is approximately 3,200 mm, highest being in the months of July to September with an average monthly temperature of 16–26°C. The prospective hospital based study was conducted at the Manipal Teaching Hospital, Pokhara for a period of 6 years (October 1999 – May 2005). This institute hospital is the only tertiary care centre at Pokhara which caters services to the people of the Kaski and its surrounding districts.

Three stool samples were collected in waterproof screw capped plastic containers from each of the 2062 (1204 males and 858 females) children below 15 years, who provided informed written consent/child assent. The consent was obtained either from the parents of the children or the adult guardians (e.g. school teacher) accompanying the children. The children were either admitted to the hospital or visited the out patient department. Three stool samples were also collected from 220 children (126 males and 96 females) who came for medical care at different medical camps as a part of community survey in all the 8 districts, including Kaski. These children had not visited hospital for at least 2 months, and had not received any antihelminthic treatment. The sample size was not uniform from every camp. The samples were processed immediately using direct method. Formol – Ether concentration method was used to increase the yield of helminth eggs [21]. The Kato-Katz technique was used to measure the intensity of infection since it provides an accurate measure of the number of eggs present [21,22]. Intensity of infection was described as range of infection, arithmetic mean and geometric mean. Infection intensity was further classified as light, moderate and heavy according to WHO criteria [1].

Every year was divided into 4 seasonal patterns: January – March (Spring), April – June (Summer), July – September (Monsoon) and October – December (Winter) according to the local meteorological office.

Statistical analysis was done using SPSS 15 for Windows. Comparison of categorical variables was carried out with Chi-square continuity correction or Fischer's exact tests. Normally distributed data were analyzed using Student's *t *– test and analysis of variance (ANOVA). Association among soil-transmitted helminth species was investigated by 2 × 2 contingency table, for which the Chi-square statistics was calculated. Values of p < 0.05 were taken as significant. The study received approval from the institutional ethical board. Quality check on our methodology for egg counting was maintained by examining each specimen by three different staff members and if the variation in the results of microscopy was < 10%, it was taken into consideration [1].

## Results

The children were divided into two age groups: 0–5 years (pre-school) and 6–15 years (school-age). Study group comprises 1099 preschool and 963 school-age children and control group comprises 108 preschool and 112 school-age children.

The overall prevalence of the geohelminth infection was 9.2% (189/2062) in children attending hospital whereas 17.7% (39/220) in children in community. In the study group, 83 (43.9%) preschool and 106 (56.1%) school-age children had infection. The prevalence of geohelminth infection in the preschool children was 7.6% (83/1099) in hospital and 14.8% (16/108) in community whereas in school-age children, 11.0% (106/963) in hospital and 20.5% (23/112) in community.

The commonly detected geohelminths were *A. lumbricoides*, *T. trichiura*, hookworm and *S. stercoralis*. Prevalence of geohelminth infection – single and mixed (presence of two or more parasite species) in pre-school (0–5 years) and school-age (6–15 years) groups, in hospital and community set up is shown in Table 1. Seventy two (86.7%) preschool children had single and 11 (13.3%) had mixed infection (2 species) whereas 88 (83.0%) school-age children had single and 18 (17.0%) had mixed infection (2 species). There was no mixed infection from community. Overall, the maximum number of infections was by *A. lumbricoides*, followed by hookworm, *T. trichiura*, and *S. stercoralis *in both age groups.

**Table 1:** Distribution of geohelminths by age

**Geohelminths**	**0 – 5 years**		**6 – 15 years**	
	**Hospital**	**Community**	**Hospital**	**Community**
***A. lumbricoides***	28(33.7)	6(37.5)	43(40.56)	13(56.5)
*T. trichiura*	18(21.7)	3(18.7)	18(16.9)	7(30.4)
Hookworm	24(28.9)	4(25.0)	22(20.7)	6(26.1)
*S. stercoralis*	2(2.4)	-	5(4.7)	-

Mixed infection				

AL + TT	3(3.6)	-	7(6.6)	-
A + H	4(4.8)	-	6(5.7)	-
H + TT	4(4.8)	-	5(4.7)	-
Total	83	13	106	26

AL = *A. lumbricoides*, TT = *T. trichirua*, H = Hockworm

Note: Percentages are given in parenthesis.

Intensity of infection was measured as egg per gram in children in hospitals from 8 districts in Western Nepal over 6 years (Table 2). The comparative analysis of the intensity of geohelminths in children attending the hospital from all the districts showed no statistical difference (p > 0.05). The worm load, however, showed a decrease in Kaski district during the study period. Yearly distribution of the intensity of geohelminth infection with regard to light, moderate and heavy infection is depicted in Table 3. Although the overall prevalence of infection by any geohelminth showed an increase during 1999–2005, the percentage of heavy infection has decreased in successive years.

**Table 2:** The study population surveyed in the Western Nepal and the intensities of geohelminths

**District**	**Study year**	**Total no. of stool examined (n = 2062)**	**No. (%) of positive stool (n = 189)**	**Intensity (epg)**					
				
				***A. lumbricoides***		***T. trichiura***		**Hookworm**	
				
				**Range**	**AM/GM**^#^	**Range**	**AM/GM**^#^	**Range**	**AM/GM**^#^
Kaski	1999	103	09(8.7)	356–88596	31532/554	94–1034	594/399	4912–6012	5530/5510
	2000	127	12(9.4)	578–99624	26505/897	112–1212	734/492	4656–8564	6632/6431
	2001	173	12(6.9)	412–110124	41262/3979	102–924	523/371	4438–5172	4877/4868
	2002	226	15(6.6)	778–112342	48112/3888	88–1278	586/404	2516–5554	3284/3126
	2003	299	21(7.0)	612–6778	2851/2071	48–720	269/169	1302–4122	2509/2316
	2004	353	24(6.8)	824–4654	2398/1953	24–338	117/81	226–2986	1111/866
	2005*	95	10(10.5)	394–4654	1408/976	28–234	131/80	232–1016	568/475

Lamjung	2002	25	03(12.0)	9058	-	264	-	1058	-
	2003	31	04(12.9)	572–4632	2602/1627	24	-	4326	-
	2004	38	05(13.2)	498–3774	2136/1370	28–178	103/70	778	-
	2005*	21	03(14.3)	216	-	156	-	3712	-

Manang	2002	27	01(3.7)	4536	-	-	-	-	-
	2003	37	04(10.8)	432	-	578	-	2002–4026	3014/2839
	2004	40	05(12.5)	288–7800	3400/1680	222	-	978–4128	2553/2009
	2005*	19	02(10.5)	616	-	78	-	-	-

Myagdi	2002	15	02(13.3)	456	-	216	-	-	-
	2003	36	03(8.3)	162–4566	2364/860	234	-	-	-
	2004	45	05(11.1)	24–4694	2460/669	198	-	3766–4138	3952/3947
	2005*	16	03(18.8)	132	-	-	-	1216–3004	2110/1911

Parrbat	2002	11	03(27.2)	1738	-	308	-	4778	-
	2003	23	04(17.4)	2774–5788	4281/4006	554	-	3126	-
	2004	22	06(27.3)	468–3118	1596/1205	172	-	2112–4664	3388/3138
	2005*	16	02(12.5)	138–654	396/300	-	-	-	-

Baclung	2003	25	02(8.0)	2712	-	416	-	-	-
	2004	54	05(9.3)	492–1106	794/753	-	-	886–3626	2256/1792
	2005*	36	03(8.3)	146–1976	1061/537	-	-	5406	-

Syangja	2004	58	06(10.3)	178–1576	707/484	-	-	566–4010	1934/1406
	2005*	23	06(26.0)	224–1038	609/508	-	-	418–4116	1919/1281

Tanahu	2004	43	03(7.0)	258–4762	2494/1446	-	-	-	-
	2005*	25	06(24.0)	276–1274	764/639	172	-	322–5236	3069/1832

• * Up to May, 2005

^•# ^AM = Arithmetic mean, GM = Geometric mean

• Note: Percentages are given in parenthesis.

**Table 3:** Distribution of geohelminths by year

**Year**	**No. of samples studied n = 2062**	**No. of positive samples number (%) Total 189**	**Mixed infection Total 29**	**Percent distribution**														
				
				***A. lumbricoides***					***T. trichiura***					**Hookworm**				
				
				Range	AM/GM	L	M	H	Range	AM/GM	L	M	H	Range	AM/GM	L	M	H
1999	103	09(8.7)	2	356 – 88596	31532/554	33.3	33.3	33.3	112 – 1212	460/128	66.6	33.3	-	4656 – 8564	6632/1121	-	-	100.0
2000	127	12(9.4)	3	578 – 99624	26505/897	42.8	14.2	42.8	102 – 924	523/108	100.0	-	-	4438 – 5172	4877/3284	-	-	100.0
2001	173	12(6.9)	6	412 – 110124	41262/3979	37.5	25.0	37.5	88 – 1278	614/175	80.0	20.0	-	2516 – 5554	2632/1169	-	80.0	20.0
2002	304	24(7.9)	6	456 – 112342	42783/4708	84.6	15.3	-	48 – 720	402/177	100.0	-	-	1302 – 4122	2327/1432	33.3	44.4	22.2
2003	451	38(8.4)	5	162 – 6778	2477/1251	95.8	4.1	-	24 – 578	375/155	100.0	-	-	226 – 4664	1879/1191	57.1	23.8	19.0
2004	653	59(9.0)	5	24 – 4762	2033/352	100.0	-	-	28 – 234	78/69	100.0	-	-	232 – 5236	3008/2054	63.6	18.1	18.1
2005*	251	35(13.9)	2	138 – 4654	368/212	100.0	-	-	78 – 172	-	100.0	-	-	412 – 912	530/47	100.0	-	-

• *Up to May

• Intensities are defined according to WHO Criteria (Montresor A *et al*, 1998)

• Note: Percentages are given in parenthesis.

• L – Light; M – Moderate; H – Heavy infection.

Variations in the intensity of geohelminth infections according to gender, age and seasonal changes are described in Table 4. There was significant differences (p = 0.008, continuity correction 6.95) in intensities by age in hospital as well as in the community and the school-age children had the highest egg counts. However, no significant difference was detected (p = 0.093, continuity correction 2.826) with relation to gender. School-age children were infected heavily by *A. lumbricoides *and hookworm, both in the hospital and community. For *T. trichiura*, both the age groups had a light infection pattern. However, there was no significant seasonal variation (p = 0.424).

**Table 4:** Prevalence and intensities of geohelminths, by gender, age and season in the children attending hospital in Western Nepal

	**Total no. of cases studied**	**Total no. of Positive cases (n = 189)**	**Cumulative Prevalence**	**Prevalence (%)**			**Intensity**					
				
				**AL**	**TT**	**HW**	**AL**		**TT**		**HW**	
							
							**Range**	**AM/GM**	**Range**	**AM/GM**	**Range**	**AM/GM**
**Gender**	**Males (1204)**	99	8.2	37.3	21.2	25.2	146 – 112342	10572/4654	28 – 1278	454/268	886 – 8564	3732/2616
	**Females (858)**	90	10.5	37.7	16.6	23.3	24 – 110124	12505/3233	24 – 924	518/307	232 – 5666	1443/1291
**Age**	**0 – 5 y (1099)**	83	7.5	33.7	21.6	28.9	146 – 66428	4008/1080	28 – 924	224/119	226 – 5486	2158/1359
	**6 – 15 y (963)**	106	11	40.5	16.9	20.7	24 – 112342	18696/2515	24 – 1278	146/146	232 – 8564	3112/2664
**Season**	**Summer (641)**	65	10.1	39.4	22.5	32.3	258 – 99624	9820/2339	78 – 1278	566/384	226 – 7822	3138/2234
	**Monsoon (994)**	93	9.3	39.0	23.8	30.4	24 – 112342	16110/1984	98 – 1034	474/337	1022 – 8564	3765/2804
	**Winter (304)**	21	6.9	55.5	14.8	18.5	138 – 88596	13228/1608	48 – 156	237/122	684 – 5172	4092/3625
	**Spring (123)**	10	8.1	53.3	13.3	33.3	356/2876	1357/841	ND	ND	ND	ND

Note: AL – *Ascaris lumbricoides*; TT – *Trichuris trichiura*; HW – Hookworm

## Discussion

Geohelminth infections are prevalent in many countries, especially in tropics and subtropics and continue to be of importance due to their high prevalence and effects on morbidity in the population [23]. To our knowledge, this is one of the most descriptive hospital based geohelminth studies from the South East Asian region, which has been carried out to determine the prevalence and intensity in children at a high altitude. Three aspects enhance the importance of this study: (1) This is one of the few and most exhaustive studies over a period of 6 years on geohelminths carried out at high altitude, (2) our study mainly concerns children of Western Nepal who usually have low nutritional uptake and high energy expenditure, and (3) this study measures the prevalence, infection intensity and changes in the intensity over years.

The infection rate among children in hospital (9.2%) and in the community (17.7%) from our study was not high as reported in other studies [24-27], although none of these studies were carried out at high altitude. However, a study from the plain lands of Sri Lanka showed low prevalence, ranging from 2.0–5.4% in 1997–99 [28]. Despite inadequate sanitation, poor hygiene (indiscriminate defecation), inaccessible health care and poverty of this region, the results obtained in our study are markedly lower than the other developing as well as developed countries [24-27]. This may be due to execution of proper heath control measures in Kaski district like municipality tap water, deworming programmes for school-age children and health education regarding treatment of well water. A study from the high altitude of Bolivia showed the prevalence rates of 18.0% and 23.8% in school and community surveys [29], which are comparable to our findings.

Few authors have suggested that the altitude along with its unique environmental characteristics have a prominent effect over the infection pattern in human beings [30-32]. Western Nepal is located at a high altitude where the average monthly temperature is 16–26°C with high evapotranspiration and intense solar radiation. These characteristics are in additional to the typical lower partial oxygen pressure in ambient air at high altitude. Geohelminths like *A. lumbricoides *and *T. trichiura *have similar life cycles and are transmitted by eggs which require optimum environmental conditions and time to embryonate into the infective stage, once voided in the host faeces. The moisture in the soil during monsoon and late summer help in the molding within the eggs. The presence of autochthonous hookworm and *S. stercoralis *is worth mentioning, since similar high altitude study from Bolivia highlighted the absence of these two nematodes in their study [29]. A 12-month-study in slum areas found significant reductions of the prevalence and the intensity of *A. lumbricoides *and *T. trichiura *infections [33], where seasonal variation was considered as one of the probable explanations [34]. However, the lack of significant association with seasonal changes in our case strengthens the findings in the Bolivian study, that the transmission is not always seasonal. However, results from a hospital based survey do not warrant significance and additional community surveys in this geographical area would help understand this pattern.

The comparative high infection load of both *A. lumbricoides *and hookworm in children does not correspond to the intensity pattern of *T. trichiura*, which is always light. This finding may perhaps be related with the difference in egg viability of this species [31,32]. The prevalence of *A. lumbricoides *and *T. trichiura *is also associated with age. *A. lumbricoides *infection is highest among children and decreases with age whereas infection of *T. trichiura *rises during childhood and adolescence and an increase is seen till old age [35]. A study from rural Honduran communities observed that tap water, as a source of drinking water might be responsible for the high prevalence of *A. lumbricoides*, but not infection with *T. trichiura*, whereas heaviest infection with both the species was encountered in those communities, where people had to walk long distance to get water [36]. However, the reason for this association was not certain. In Pokhara, almost all the houses are supplied with municipal tap water, which might have a coincidental association with the high load of *A. lumbricoides *as compared to *T. trichiura*. Results obtained from three samples for an individual were more significant than compared to single specimen analysis. Of the three samples collected, occasionally in one sample no geohelminth was detected but results were positive for the remaining samples. This may be due to intermittent shedding of geohelminth eggs/larva in stool [17].

Many observers have indicated a definite relation between gender and the transmission of infection [29]. In our study, both males and females have a more or less similar infection rate. The infection rate could depend on the role giving one or other sex greater exposure to the contaminated soil. The significantly increased intensity of geohelminth infection in school-age children can be explained by the fact that they are more exposed to unhygienic practices, contaminated foods and water. Children below 5 years are usually under direct supervision of the parents and consume home prepared foods. Although the prevalence of infection is more in the community level, the intensity is higher in those children who visited hospital. Light infections in community children are mostly asymptomatic, whereas moderate to heavy infection might give rise to clinical symptoms. Since the survey is hospital based, it does have many drawbacks. The findings suggest that there might be an overall increase in prevalence of geohelminth in this geographical area. However, few children attended hospital with heavy infection than in the previous years, which might be an effect of regular prescription of antihelminthic drugs to the children from the hospital. The true picture might be different, which can only be assessed with mass community survey in this area. It would also be interesting to study the association of spatial patterns and environmental risk factors including the elevation, temperature and annual rainfall from each district with that of helminth infections. The treatment follow up of the children was not done due to poor turn up, which could have provided the evidence of the effectiveness of the deworming programme.

## Conclusion

The Kaski district (Pokhara) in Western Nepal can be classified as Grade III according to WHO grading system [1]. However, a hospital-based study may not be appropriate to draw a conclusion regarding community prevalence and label it an endemic area. The study would be a healthy base for future community epidemiological studies of geohelminthic infection in Nepal.

## Competing interests

The author(s) declare that they have no competing interests.

## Authors' contributions

CM proposed the study and surveillance plan, analyzed data and wrote the manuscript

GW took active part in surveillance and data collection, and analyzed data

KC analyzed data and wrote the manuscript

BVS analyzed data and done the statistical evaluation

PGS provided guidance to study and surveillance, and edited the manuscript

All authors read and approved the final manuscript

## Pre-publication history

The pre-publication history for this paper can be accessed here:



## References

[B1] Montresor A, Crompton DWT, Hall A, Bundy DAP, Savioli L Guidelines for the evaluation of soil-transmitted helminthiasis and schistosomiasis at community level. Geneva, Switzerland: World Health Organization WHO/CTD/SIP/981.

[B2] WHO Prevention and Control of Intestinal Parasitic Infections. Report of a WHO Expert Committee Geneva, Switzerland: World Health Organization, Technical Report Series, 1987, no749.

[B3] Callender JEM, Grantham-McGregor S, Walker S, Cooper ES (1992). Trichuris infection and mental development in children. Lancet.

[B4] Cooper ES, Whyte-Allenge CAM, Finzi-Smith JS, MacDonald TT (1992). Intestinal nematode infections in children: the pathophysiological price paid. Parasitology.

[B5] Crompton DWT, Montresor A, Nesheim MC, Savioli L (2003). Controlling disease due to helminth infection.

[B6] Savioli L, Bundy D, Tomkins A (1992). Intestinal parasitic infections: a soluble public health problem. Transactions of the Royal Society of Tropical Medicine and Hygiene.

[B7] Nokes C, Bundy D (1994). Does helminth infection affect mental processing and educational achievement?. Parasitology Today.

[B8] Levav M, Mirsky AF, Schantz PM, Castro S, Cruz ME (1995). Parasitic infection in malnourished school children: effects on behaviour and EEG. Parasitology.

[B9] Booth M, Bundy DAP, Albonico M, Chwaya HM, Alawi KS, Savioli L (1998). Associations among multiple geohelminth species infections in schoolchildren from Pemba Island. Parasitology.

[B10] Ezeamama AE, Friedman JF, Olveda RM, Acosta LP, Kurtis JD, Mor V, McGarvey ST Functional Significance of Low-IntensityPolyparasite Helminth Infections in Anemia. J Infect Dis.

[B11] Rai SK (1980). Helminthic infestation in local Nepalese people. Ankur, Institute of Medicine Central Campus (Nepal).

[B12] Reily C (1980).

[B13] Estevez EG, Levine JA, Warren J (1983). Intestinal parasites in a remote village in Nepal. J Clin Microbiol.

[B14] Rai SK, Nakanishi M, Upadhyay MP, Rai CK, Hirai K, Ohno Y (1998). Effect of intestinal helminth infection on some nutritional parameters among rural villagers in Nepal. Kobe J Med Sci.

[B15] Bordignon GP, Shakya DR, Crompton DWT, Montresor A, Nesheim MC, Savioli L (2003). A deworming programme in Nepal supported by the World Food Programme. Controlling disease due to helminth infections.

[B16] Crompton DWT (1992). Ascaris and childhood malnutrition. Trans Royal Soc Trop Med Hyg.

[B17] Kang G, Mathew MS, Rajan DP, Daniel JD, Mathan MM, Mathan VI, Muliyli JP (1998). Prevalence of intestinal parasites in rural Southern Indians. Trop Med Int Health.

[B18] Naish S, McCarthy J, Williams GM (2004). Prevalence, intensity and risk factors for soil-transmitted helminth infection in a South Indian fishing village. Acta Tropica.

[B19] Holland CV, Taren DL, Crompton DWT, Nesheim MC, Sanjur D, Barbaeu I, Tucker K, Tiffany J, Rivera G (1988). Intestinal helminthiases in relation to the socioeconomic environment of Panamanian children. Soc Sci Med.

[B20] CARE Nepal (1995). A study of the evaluation of home gardening programme in Bajura and Mahottari districts.

[B21] Gillespie SH, Gillespie SH, Pearson RD (2001). Intestinal Nematodes. Principles and Practice of Clinical Parasitology.

[B22] Ash LR, Orihel TC, Savioli L (1994). Bench aids for the diagnosis of intestinal parasites.

[B23] WHO (1981). Intestinal protozoan and helminthic infections: report of a WHO Scientific Group. WHO Tech Rep Ser.

[B24] Hillyer GV, Soler De Galanes M, Lawrence S (1990). Prevalence of intestinal parasites in a rural community in north-central Puerto Rico. Bol Assoc Med PR.

[B25] Ferrari JO, Ferreira MU, Camargo LM, Ferreira CS (1992). Intestinal parasites among Karitiana Indians from Rondonia State, Brazil. Rev Inst Med Trop Sao Paulo.

[B26] Oberg C, Biolley MA, Duran V, Matamala R, Oxs E (1993). Intestinal parasites in the riverside population of Villarrica Lake Chile. Bol Chil Parasitol.

[B27] Ferreira CS, Ferreira MU, Nogueira MR (1994). The prevalence of infection by intestinal parasites in an urban slum, Sao Paulo, Brazil. J Trop Med Hyg.

[B28] Behnke JM, Clercq DD, Sacko m, Gilbert FS, Ouattara DB, Vercruysse J (2000). The epidemiology of human hookworm infections in the southern region of Mali. Trop Med Int Health.

[B29] Flores A, Esteban JG, Angles R, Mas-Coma S (2001). Soil-transmitted helminth infections at very high altitude in Bolivia. Trans Royal Soc Trop Med Hyg.

[B30] Meakins RH, Harland PSEG, Carswell F (1981). A preliminary survey of malnutrition and helminthiasis among school children in one mountain and one lowland Ujamaa village in northern Tanzania. Trans Royal Soc Trop Med Hyg.

[B31] Crompton DWT, Nesheim MC, Pawlowski ZS (1989). Ascaris and its public health significance.

[B32] Appleton CC, Gouws E (1996). The distribution of common intestinal nematodes along an altitudinal transect in KwaZulu – Natal, South Africa. Ann Trop Med Parasitol.

[B33] Hadju V, Stephenson LS, Mohammed HO, Bowman DD, Parker RS (1998). Improvements of growth, appetite, and physical activity in helminth-infected school boys 6 months after single dose of albendazole. Asia Pacific J Clin Nutr.

[B34] Cabrera BD (1984). Reinfection and infection rates of ascariasis and trichuriasis among school children in relation to seasonal variation in the Philippines. South Asian J Trop Med Pub Hlth.

[B35] Verle P, Kongs A, De NV, Thieu NQ, Depraetere K, Kim HT, Dorny P (2003). Prevalence of intestinal parasitic infections in northern Vietnam. Trop Med Int Health.

[B36] Smith HM, DeKaminsky RG, Niwas S, Soto RJ, Jolly (2001). Prevalence and intensity of infections of *Ascaris lumbricoides *and *Trichuris trichiura *and associated socio-demographic variables in four rural Hounduran communities. Mem Inst Oswaldo Cruz.

